# Attachment and relationship-based interventions for families during neonatal intensive care hospitalization: a study protocol for a systematic review and meta-analysis

**DOI:** 10.1186/s13643-020-01331-8

**Published:** 2020-03-21

**Authors:** Ah. Rim Kim, Soo-yeon Kim, Ji Eun Yun

**Affiliations:** 1 Department of Nursing, Far East University, 76-32 Daehak-gil, Gamgok-myeon, Eumseong-gun, Chungbuk 27601 Republic of Korea; 2 Department of Nursing, Daegu Haany University, Daegu, Republic of Korea; 3 Division for Healthcare Technology Assessment Research, National Evidence-based Healthcare Collaborating Agency, Seoul, Republic of Korea

**Keywords:** Preterm infants, Mothers, Neonatal intensive care, Attachment, Interventions, Hospitalization

## Abstract

**Background:**

Attachment in the parent-infant dyads is fundamental for growth and development of children born prematurely. However, the natural process of attachment is interrupted just after preterm birth, and emotional and physical detachment, limited social interaction, and a traumatic, technologically heavy environment in a neonatal intensive care unit (NICU) may result in impaired attachment or bonding. To our knowledge, few studies have evaluated the effectiveness of interventions aimed at enhancing attachment, bonding, and relationships between parents and their preterm infants during the infant’s hospitalization in the NICU. This study aims to perform a comprehensive systematic review and a meta-analysis survey of the effects of attachment- and relationship-based interventions in the NICU.

**Method:**

A comprehensive literature review will be conducted in the following databases: MEDLINE, CINAHL, PubMed, EMBASE (OVID), Scopus, PsycINFO (OVID), Cochrane Database of Systematic Reviews, Cochrane Central Register of Controlled Trials (CENTRAL), and Web of Science. Selected studies will be published in English, in the last 20 years, from 1999 onwards. All studies of randomized controlled trials (e.g., parallel groups, cluster) will be included. We will consider studies evaluating attachment- and relationship-based interventions (e.g., skin-to-skin contact, parental involvement in infant care) versus a comparator (standard of care). The primary outcome will be maternal attachment. Secondary outcomes will include infants’ growth and development, family health, and parenting experience. Data extraction from eligible studies will be conducted independently by two experts who will compare their data. The Cochrane risk of bias tool will be applied to the selected studies. If data permits, we will conduct random effects meta-analysis where appropriate. Subgroup and additional analyses will be conducted to explore the potential sources of heterogeneity considering gender of parents, infants’ sex, and gestational age. Data synthesis will be carried out using the RevMan 5.3 software. Publication bias will be assessed with the graphical funnel plot method and the Egger test. The quality of the evidence will be rated using the methods of the Grades of Recommendation Assessment, Development and Evaluation (GRADE) Working Group.

**Discussion:**

The results of this systematic review will discuss the types of attachment- or relationship-based interventions that are effective for facilitating family health outcomes and the babies’ growth and development and will contribute to establishing new evidence in neonatal and family-centered care by providing scientific guidance for clinical practice and further research.

**Systematic review registration:**

PROSPERO CRD42019145834

## Background

The natural process of parent-infant attachment can be interrupted due to diverse factors, such as the infant’s illness, parental psycho-emotional distress, and NICU admission just after birth (i.e., preterm birth and emergent cesarean section due to low Apgar scores) [1]. In fact, one longitudinal study revealed that infants who were hospitalized in the NICU showed a higher prevalence (6.1 times) of disorganized attachment at the age of 3 years [2]. Although advances in neonatal technology have allowed preterm or low-birth-weight infants with special needs to survive more than ever before, impaired attachment or bonding in the NICU owing to alteration in parental role, post-traumatic shock of families, and possible neurodevelopmental disabilities in preterm infants still remains an issue [3].

The interpretation of the attachment process comes from a developmental perspective on human life. Bonding to the primary caregiver is fundamental to growth and development in children; the caregiver is identified as a “secure base” from which to explore the external environment after birth [4]. In the neurobiological underpinnings of human attachment, the processes involved in the maturation of the brain and activation of neuroendocrine systems operate according to the mother-offspring relationship through attachment-related behaviors, especially during the first 2 years of life [5]. *Attachment* refers to the connection between mother and infant, although *bonding* is described as the “emotional tie from parent to infant” [6]. The meaning of bonding is more focused on the mother’s views toward the relationship with the child, which can be constituted by behavioral domains (e.g., parent-infant proximity, and physiological domains (e.g., oxytocin levels) [7]. Even though they should be defined separately in research, attachment and bonding have been used interchangeably in diverse contexts [8]. Therefore, this review will examine studies on attachment-, bonding-, and relationship-based interventions.

Since 1999, researchers have considered attachment in the NICU to be an individualized process, contradicting earlier work by Rubin, which suggested that attachment occurs autonomically [9]. Attachment between the parent and preterm infant can be facilitated through protective factors such as nurturing touch, close proximity, caregiving, sensitivity to the infant’s cues (recognition and interpretation), and responsiveness to the infant’s needs (identification of needs and meeting of needs) [10, 11]. However, the early separation between the parent and preterm infant and a traumatic technological environment interrupt the complex attachment process [12]. There is consensus that the infant-, NICU environmental-, and family-related factors that influence the parent-preterm infant attachment, bonding, and relationship are neonatal characteristics (i.e., prematurity, gestational age, and congenital defects with severe complications) [4, 13], parental sensitivity [11], caregiving [14, 15], parent-infant closeness and interaction [16, 17], supportive nursing care [18], parental mental health and emotional distress [19, 20], and tactile stimulation and skin-to-skin contact [21, 22]. Hence, interventions aimed at supporting or modifying these factors during the NICU stay should help establish parent-infant attachment.

Previous systematic reviews and review protocols on NICU interventions have included preterm infant health outcomes (e.g., neurodevelopment [3], microbiome [23], speech and communication skills [24], and health severity) [25] and family outcomes (e.g., parenting sensitivity [11], stress and parenting stress) [25]. A comprehensive review of the effects of attachment-based interventions on prenatal attachment will be performed [26]; however, unfortunately, no studies have specifically evaluated the effectiveness of NICU interventions aimed at promoting attachment, bonding, or the relationship between the parent and preterm infant during the infant’s hospitalization. Identifying interventions that effectively promote attachment in NICU families following preterm birth may foster good short- and long-term developmental health outcomes and promote maternal and paternal well-being. The results of this review will provide guidance for the development and implementation of attachment- and relationship-based interventions in neonatal clinical practice and for tailored protocols for family- and attachment-based interventions in specific contexts.

This systematic review aims to assess the effects of attachment- and relationship-based interventions designed to facilitate parental proximity to the preterm/low birth weight infants and of mother–infant interaction during NICU hospitalization on infants’ growth and development, maternal and family health outcomes, treatment/clinical outcomes, and parenting experience. This systematic review is guided by the following questions. Are there attachment or relationship-based interventions that are effective for the treatment of babies hospitalized in the NICU? What is the effectiveness of attachment- or relationship-based interventions for NICU babies and their mothers on the developmental health outcomes of premature infants, maternal health, and parenting experiences?

## Methods/design

The present protocol has been registered within the PROSPERO database (registration number CRD42019145834) and is being reported in accordance with the reporting guidance provided in the Preferred Reporting Items for Systematic Reviews and Meta-Analyses Protocols (PRISMA-P) statement (see Additional file 1: PRISMA-P 2015 checklist) [27].

### Eligibility criteria

Studies will be selected according to the following criteria: study design, participants, interventions and comparators, outcome(s) of interest, and context.

#### Study design

We will include experimental studies, such as randomized controlled/clinical trials (RCTs) and cluster RCTs. When searching, studies will be excluded if (1) the randomization process was not random and (2) the study design was quasi-experimental (without randomization or comparative group), descriptive, and case-controlled. The highest level of evidence provided by the RCT design can assure the reliable findings and strongest inferences in this systematic review.

#### Participants

Studies that included parents and their infants born at or 36 weeks of gestation or weighing less than 2500 g will be considered. We will include studies of the parents of all age groups, ethnicities, and geographical areas.

#### Interventions and comparators

Interventions intended to improve relationships or attachment in infant–parent dyads, referred to as attachment/bonding/relationship-based interventions, will be considered. All interventions, aimed either at premature/low birth weight infants or parents, implemented during NICU hospitalization will be included. Examples include interventions designed to promote mother–infant interaction, parent–infant attachment (attachment-oriented interventions, such as skin–to–skin contact/kangaroo care), and/or those designed to facilitate maternal proximity, parental involvement in baby care, and parenting quality (maternal role/mothering [parenting interventions]). Multi-component interventions will not be included because these will not enable easy identification of the component that had more influence on infants’ and familial outcomes and will hinder meta-analysis, if even applicable. If needed, we will contact the original authors to gain detailed information on the interventions. Comparator groups of any type, such as a usual care group or non-exposed control/different intervention group, will be included in this review.

#### Outcomes of interest

The primary outcome will be the mother-infant relationship/attachment/bonding as an outcome evaluated with a standardized screening tool, instrument, or scale during the babies’ NICU hospitalization. Examples of standardized self-report tools, scales, and instruments measuring attachment or the relationship of mother–infant dyads are the Maternal Attachment Inventory [28], Postpartum Bonding Questionnaire [29], Maternal Postpartum Attachment Scale [30], Maternal Feelings Questionnaire [31], and Mother-to-Infant Bonding Questionnaire [32]. The secondary outcomes will be infants’ growth and development (i.e., neurodevelopmental, behavioral, and cognitive-emotional problems) and treatment/clinical outcomes (i.e., weight gain, complications, physiologic stability, and hospitalization period). Additional outcomes of interest are maternal and family health outcomes (i.e., psychosocial distress and postpartum depression) and parenting/nurturing experience (i.e., parenting stress, knowledge of infant cues, and maternal sensitivity/responsiveness). Outcomes will not be used to set inclusion or exclusion criteria.

#### Context

There will be no restrictions in regard to geographical area or context.

#### Further inclusion criteria

Selected studies will be limited to those written in English, with a full text available, and will have been published in the last 20 years, from 1999 onwards. We will restrict the searches from 1999, the publication year of the article that reported new perspectives on the attachment process between a mother and her infant, as not an automatic, but an individualized one in the NICU [9].

#### Exclusion criteria

Newspaper/book articles, editorials, protocols, and conference proceedings will not be included in the review due to the possibility of subjectivity and publication bias. Articles about intervention studies with methodologically week, and duplication of previous reported dataset will be excluded from the review.

### Information sources and search strategy

The following electronic databases will be used to search for relevant studies using MeSH/Thesaurus terms: MEDLINE, PubMed, EMBASE (OVID), CINAHL, Scopus, Cochrane Database of Systematic Reviews, Cochrane Central Register of Controlled Trials (CENTRAL), PsycINFO (OVID), and Web of Science. A comprehensive list of keywords and medical subject heading (MeSH) terms will be incorporated and combined for each part of the PICO (see Additional file 2: Search strategy). Diverse combinations of keywords which will be combined by Boolean operators AND and OR. Available keywords and Boolean operators will be further considered for a suitable change of strategy in particular database. We will also review the Scopus database to screen for trials in conference proceedings and search gray literature sources, such as ProQuest, Dissertations and Theses database, OpenGREY, and the Grey Literature Report. We will likewise search online trial registers, such as the clinicaltrials.gov website, and the World Health Organization International Clinical Trials Registry Platform (http://apps.who.int/trialsearch), to identify other ongoing/potential and recently completed studies.

### Study selection

All retrieved studies initially included for this systematic review will be imported to the EndNote© program. All duplicates of studies will be identified and subsequently removed in the program after being screened by two reviewers independently and then compared to check the correct deletion of studies. These two separate reviewers will assess the eligibility of the remaining studies as follows. All studies’ titles and abstracts will be identified to ensure that the studies meet the eligibility criteria in the first stage. Second, all full-text papers selected in the first stage will be reviewed thoroughly to assess their suitability for inclusion in this systematic review. An agreement will be reached after discussion with the two reviewers during the screening process (i.e., deleting duplicates and evaluating titles/abstracts and then full-text reports). A third expert, as an arbiter, will be consulted in the case where agreements cannot be reached. The number of studies screened, included, and excluded, and the reasons for ineligibility and exclusion, will be documented in an adapted PRISMA flow chart [33].

### Data extraction

Data extraction will be conducted independently by a minimum of two reviewers, followed by a comparison of output. A pilot test for data extraction with at least five studies will be conducted to develop a predefined data extraction form. Collected data in the form will include the following: study title, trial registry number, authors and publication year, study design, country of study, number of centers included, sample size, description of sample (infants’ gestational age, birth weight, sex, age at assessment, order of birth, maternal age at birth, household income, marital status, education level, and employment status), intervention (name, hospital setting, who delivered it, frequency, timing/onset, duration, follow-up, dose, and contents), timing of evaluation, and outcome, including the definition of attachment/bonding/mother–infant relationship as well as the screening tools, scales, instruments, or tests used to measure this outcome. All extracted data will be handled in the RevMan 5.3 software (The Cochrane Collaboration, Copenhagen, Denmark).

### Risk of bias assessment

Two reviewers will independently assess the risk of bias in the included studies, with any disagreements resolved by the involvement of a third expert or by discussion to reach consensus. The quality of individual studies will be evaluated using the Cochrane risk of bias 2.0 tool for randomized controlled trials [34]. This tool is designed to classify the risk of bias in each article as low, high, or some concerns, in which the former is regarded as a high-quality study. We will use these results to elucidate the rigor of the involved articles, while we will not include studies with a high risk of bias.

### Data synthesis

We will describe clinical heterogeneity (i.e., samples, interventions, timing of evaluation, and outcome measurement) and methodological heterogeneity (risk of bias) in data synthesis. Bonding, attachment, parental confidence or sensitivity, and infant’s gestational age and birth weight of the intervention and control group will be compared with a standardized mean difference, with 95% confidence interval (CI). Dichotomous outcome data (i.e., attachment security/quality) will be presented as a risk ratio (RR) with 95% CI.

A priori, meta-analyses will be conducted using the random effects model since clinical and epidemiological heterogeneity is expected. The random effects model assumes the treatment effects follow a normal distribution, considering both within-study and between-study variation. Forest plots will be presented to visualize pooled estimates and the extent of heterogeneity among studies. Higgin’s *I*^2^ statistic will also be used to assess heterogeneity of the studies with the following criteria: (1) 0–40%, not important heterogeneity; (2) 30–60%, moderate heterogeneity; (3) 50–90%, substantial heterogeneity; and (4) 70–100%, significant heterogeneity [27]. A random effects model and subgroup analysis will be considered for meta-analysis if a *p* value of 0.10 or smaller for the *χ*^2^ test or if moderate heterogeneity (*I*^2^ < 50%) is identified. If possible, subgroup analysis will be performed based on infants’ gestational age at birth, congenital defect, and maternal age at childbirth, as these variables have been considered as covariates in previous studies [4, 13, 35]. If heterogeneity is too substantial to be explained, thereby rendering meta-analysis impossible, we will opt to synthesize the data only narratively.

### Missing data

If necessary, the authors of the included studies will be contacted by email to discuss and seek missing data. If missing data cannot be gathered, a permutation test or the entry of replacement values will be considered [36].

### Meta-biases assessment

The impact of reporting bias will be minimized by ensuring a comprehensive search strategy for eligible studies. A graphical method of funnel plot and the Egger test for assessing publication bias among individual studies will be examined if at least ten studies are included for meta-analysis. We will screen for the following individual elements for RCTs with the Risk of Bias tool: random sequence generation, allocation concealment, blinding, completeness of outcome data, and selective outcome reporting.

### Confidence in cumulative evidence

The confidence in cumulative evidence for all outcomes will be rated through a valid approach, using the GRADE Working Group, which specifies four categories: high, moderate, low, and very low [37].

## Discussion

The results of this systematic review will aid in determining which attachment- or relationship-based interventions impact preterm infant treatment, physiological outcomes, and neurodevelopment; maternal and family outcomes such as parenting role disruption; and psycho-emotional reactions during NICU hospitalization. The results will also provide guidelines for NICU family care. By synthesizing and analyzing the existing evidence, this review will provide insights for designing effective attachment-based interventions for those who have gone through traumatic experiences due to separation from their infant after giving birth. Implementing effective interventions in the NICU (from hospital to home) will also contribute to growth, early development, and long-term health outcomes (including psychosocial, emotional, and neurobehavioral development) in children and may facilitate paternal and maternal mental health by promoting healthy transitioning to parenthood. The main analysis of this review will incorporate studies with a low or moderate risk of bias; these studies might affect the precision of the review. Publication bias can influence results, as only relevant studies written in English will be included in this review. Describing and summarizing the effectiveness of various attachment-based or relationship-based family interventions in the NICU can provide health care teams with valuable information to improve their bonding and attachment. The results from this review will underline the strongest evidence on guidelines or interventions that may support attachment, bonding, and the relationship between the parent and infant. The findings of this study will disseminate to neonatal healthcare providers in clinical practice and researchers that have contributions to improve preterm infants and family health.

Any protocol amendments made by following group discussion (ARK, SK, JEY) will be dated, and specific rationale for changes will be documented in PROSPERO. If there is a possibility to affect statistical data analyses, more suitable options will be considered by our statistical expert (JEY).

## Supplementary information

**Additional file 1..** PRISMA-P 2015 checklist.

**Additional file 2..** Search strategy.

## Data Availability

Not applicable.

## Abbreviations

NICU: Neonatal intensive care unit
GRADE: Grades of Recommendation Assessment, Development and Evaluation
PRISMA-P: Preferred Reporting Items of Systematic Reviews and Meta-analysis for Protocols
RCT: Randomized controlled/clinical trials
