# Supplementary material for: Attachment and relationship-based interventions for families during neonatal intensive care hospitalization: a study protocol for a systematic review and meta-analysis
Source: Syst Rev. 2020 Mar 21;9:61. doi: 10.1186/s13643-020-01331-8 (PMC7085138; doi:10.1186/s13643-020-01331-8)
Supplement: Supplementary file 2 — Additional file 2.. Search strategy. [file 13643_2020_1331_MOESM2_ESM.pdf]

Additional file 2. 1) Medline Search strategy

Ovid MEDLINE(R) and Epub Ahead of Print, In-Process & Other Non-Indexed Citations,

Daily and Versions(R) 1946 to September 09, 2019

Steps	Search terms and keywords	Results
1	exp infant, premature/ or exp infant, low birth weight/ or exp premature birth/	85100
2	(infant* or baby or babies or newborn* or birth* or neonatal*).mp.	1591891
3	(premature* or low birth weight*).mp.	213449
4	2 AND 3	128571
5	1 or 4	132283
6	exp Intensive Care Units/ or exp Critical Care/ or exp critical care nursing/ or exp pediatric nursing/ or exp maternal-child nursing/ or exp nurses, pediatric/	140160
7	(neonat* or newborn* or baby or babies or pediatric* or birth* or infant* or child*).mp.	3238302
8	(intensive care* or care unit* or care cent* or intensive care unit*).mp.	220746
9	7 AND 8	71651
10	6 or 9	179892
11	exp mothers/ or exp fathers/ or exp maternal behavior/ or exp mother-child relations/ or exp father-child relations/ or exp paternal behavior/	69761
12	exp Object Attachment/	14508
13	(attachment* or bond* or emotional bond* or relation* or attach* or parent* nurtur* or mother* or father* or maternal or paternal or interact* behave*).mp.	3386718
14	11 or 12 or 13	3386718
15	5 and 10 and 14	4981
16	Randomized Controlled Trials as Topic/	126306
17	randomized controlled trial/	488817
18	Random Allocation/	100342
19	Double Blind Method/	153130
20	Single Blind Method/	27263
21	clinical trial/	517804

22	clinical trial, phase i.pt	19272
23	clinical trial, phase ii.pt	31119
24	clinical trial, phase iii.pt	15487
25	clinical trial, phase iv.pt	1743
26	controlled clinical trial.pt	93272
27	randomized controlled trial.pt	488817
28	multicenter study.pt	256105
29	clinical trial.pt	517804
30	exp Clinical Trials as topic/	329910
31	or/16-30	1311950
32	(clinical adj trial$).tw.	341088
33	((singl$ or doubl$ or treb$ or tripl$) adj (blind$3 or mask$3)).tw.	165975
34	PLACEBOS/	34457
35	placebo$.tw	207101
36	randomly allocated.tw	26869
37	(allocated adj2 random$).tw.	30061
38	or/32-37	600676
39	31 or 38	1560431
40	case report.tw	293766
41	letter/	1041316
42	historical article/	353707
43	or/40-42	1673674
44	39 not 43	1525076
45	15 and 44	728
46	limit 45 to english language	701
47	limit 46 to yr="1999 -Current"	614

2) Embase (Ovid) Search strategy

Embase1976 to 2019 Week 35

Steps	Search terms and keywords	Results
1	exp prematurity/ or exp low birth weight/	137,489
2	(infant* or baby or babies or newborn* or birth* or neonatal*).mp.	1,511,254
3	(premature* or low birth weight*).mp.	249,170
4	2 AND 3	129,507
5	1 or 4	186,042
6	exp Intensive Care Units/ or exp intensive care/ or exp maternal child health care/ or exp pediatric nurse/ or exp pediatric nursing/	788,831
7	(neonat* or newborn* or baby or babies or pediatric* or birth* or infant* or child*).mp.	3,523,991
8	(intensive care* or care unit* or care cent* or intensive care unit*).mp.	435,861
9	7 AND 8	117,030
10	6 or 9	822,328
11	exp mother/ or exp father/ or exp maternal behavior/ or exp mother child relation/ or exp father child relation/ or exp paternal behavior/	155,370
12	exp Object Attachment/	11,249
13	(attachment* or bond* or emotional bond* or relation* or attach* or parent* nurtur* or mother* or father* or maternal or paternal or interact* behave*).mp.	3,525,171
14	11 or 12 or 13	3,525,591
15	5 and 10 and 14	11,586
16	Clinical Trial/	962,515
17	Randomized Controlled Trial/	569,054
18	controlled clinical trial/	464,842
19	multicenter study/	228,625
20	Phase 3 clinical trial/	42,348
21	Phase 4 clinical trial/	3,598
22	exp RANDOMIZATION/	84,318
23	Single Blind Procedure/	35,509
24	Double Blind Procedure/	165,321
25	randomi?ed controlled trial$.tw.	211,067

26	single blind$.tw.	23,620
27	double blind$.tw.	202,337
28	((treble or triple) adj blind$).tw.	1,035
29	or/16-28	1,611,942
30	Case Study/	64,141
31	case report.tw.	392,332
32	abstract report/ or letter/	1,120,797
33	Conference proceeding.pt.	0
34	Conference abstract.pt.	3,556,821
35	Editorial.pt.	631,008
36	Letter.pt.	1,085,674
37	Note.pt.	771,398
38	or/30-37	6,475,332
39	29 not 38	1,226,563
40	15 and 39	1,057
41	limit 40 to english language	1,009
42	limit 42 to yr="1999 -Current"	926

3) PsycINFO Search strategy

PsycINFO1987 to September Week 1 2019

Steps	Search terms and keywords	Results
1	exp Premature Birth/ or exp Postnatal Period/ or exp Neonatal Period/ or exp Neonatal Development/ or exp Birth/ or exp Birth Weight/	19798
2	(infant* or baby or babies or newborn* or birth* or neonatal*).mp.	145967
3	(premature* or low birth weight*).mp.	18374
4	2 AND 3	8750
5	1 or 4	22094
6	exp Neonatal Intensive Care/ or exp Intensive Care/	5337
7	(neonat* or newborn* or baby or babies or pediatric* or birth* or infant* or child*).mp.	684514
8	(intensive care* or care unit* or care cent* or intensive care unit*).mp.	16199
9	7 AND 8	6403
10	6 or 9	9402
11	exp Mothers/ or exp Mother Child Relations/ or exp Mother Child Communication/ or exp Fathers/ or exp Father Child Relations/ or exp Parent Child Relations/	83279
12	exp Object Relations/ or exp Attachment Behavior/	27994
13	(attachment* or bond* or emotional bond* or relation* or attach* or parent* nurtur* or mother* or father* or maternal or paternal or interact* behave*).mp.	942236
14	11 or 12 or 13	948349
15	5 and 10 and 14	895
16	limit 15 to english language	764
17	limit 16 to yr="1999 -Current"	764

4) PubMed Search strategy

PubMed1951 to September Week 1 2019

Steps	Search terms and keywords	Results
1	infant, newborn[MeSH] OR newborn OR neonate OR neonatal OR OR premature OR low birth weight OR VLBW OR LBW OR birth* OR child* OR infan* OR neonat*	3,502,486
2	intensive care unit, neonatal[MeSH] OR critical care[MeSH] OR mother-child nursing[MeSH] OR nurses, neonatal[MeSH] or intensive care* or care unit* or care cent* or intensive care unit*	324,659
3	1 OR 2	3,730,987
4	object attachment[MeSH] OR parent-child relations[MeSH] OR maternal behavior[MeSH] OR mothers[MeSH] OR paternal behavior[MeSH] OR fathers[MeSH]	109,500
5	attachment* or bond* or emotional bond* or relation* or attach* or parent* or maternal* or paternal* or interact* or infant-mother* or mother-infant* infant-father* or father-infant*	185
6	randomised controlled trial [pt] or randomized controlled trial [pt] OR controlled clinical trial [pt] OR randomized [tiab] OR randomized [tiab] OR randomly [tiab] OR trial [tiab]	1,263,005
7	4 OR 5	109,546
8	6 AND 7	5,509
9	3 AND 8	5,005
10	9 AND (English).lg	4,844
11	Limit 10 to yr = “1999 – 2019”	4,235

5) CINAHL Search strategy

CINAHL1939 to September Week 1 2019

Steps	Search terms and keywords	Results
1	(infant, newborn OR newborns or neonates or infants) OR (newborn or neonate or infant or baby) OR (premature infants or preterm infants or premature baby or preterm baby) OR (low birth weight or small for gestational age or low weight) OR VLBW OR LBW or Newborn or infan* or neonat*	428,264
2	(intensive care unit or icu or critical care or critical care unit) OR (neonatal intensive care unit or nicu or baby unit or newborn intensive care) OR nicu care OR neonatal intensive care	116,856
3	1 OR 2	516,069
4	(MH “attachment+”) OR (MH “bonding+”) OR (MH “relationship”)	1,194
5	attachment behavior OR (attachment or relationship or bonding) OR (attach* or bond* or relation* or interaction*) OR (emotional bond or emotional bonding) OR (infant-mother attachment OR infant-father attachment) OR (infant-mother* or mother-infant* or infant-father* or father-infant*) OR (attachment-based or relationship-based)	726,692
6	4 OR 5	726,884
7	(MH "Clinical Trials+")	266,405
8	TX allocat* random*	19,816
9	(MH "Quantitative Studies")	23,312
10	TX random* allocat*	19,816
11	(MH "Random Assignment")	56,415
12	TX randomi* control* trial*	256,383
13	TX ((singl* n1 blind*) or (singl* n1 mask*))	20,304
14	TX ((doubl* n1 blind*) or (doubl* n1 mask*))	1,046,681
15	TX ((tripl* n1 blind*) or (tripl* n1 mask*))	698
16	TX ((trebl* n1 blind*) or (trebl* n1 mask*))	11
17	TX clinic* n1 trial*	346,116
18	PT Clinical trial	86,865
19	7 OR 8 OR 9 OR 10 OR 11 OR 12 OR 13 OR 14 OR 15 OR 16 OR 17 OR 18	1,455,200
20	6 AND 19	184,618
21	3 AND 20	18,659

22	Limit 7 to yr = “1999 – 2019”	16,870
23	Limit 22 to english language	15,524

6) Cochrane Library Search strategy

Cochrane Library1944 to September Week 1 2019

Steps	Search terms and keywords	Results
1	MeSH descriptor: [Infant, Newborn] explode all trees	15,243
2	(Infant*):ti,ab,kw OR (baby):ti,ab,kw OR (babies):ti,ab,kw OR (newborn*):ti,ab,kw OR (neonat*):ti,ab,kw	71,887
3	(prematur*):ti,ab,kw OR (preterm):ti,ab,kw OR (low * weight):ti,ab,kw OR (LBW):ti,ab,kw OR (VLBW):ti,ab,kw	26,774
4	2 AND 3	16,077
5	1 OR 4	25,577
6	MeSH descriptor: [Object Attachment] explode all trees	357
7	MeSH descriptor: [Family Relations] explode all trees	2916
8	(attachment*):ti,ab,kw OR (bond*):ti,ab,kw OR (relation*):ti,ab,kw OR (interact*):ti,ab,kw OR (mother near/2 infant*):ti,ab,kw OR (parent near/2 infant*):ti,ab,kw OR (father near/2 infant*):ti,ab,kw OR (attachment-based):ti,ab,kw OR (relationship-based):ti,ab,kw	171,377
9	6 OR 7 OR 8	171,844
10	MeSH descriptor: [Clinical Study] explode all trees	150
11	(randomized controlled*):ti,ab,kw OR (randomised controlled):ti,ab,kw OR (rct):ti,ab,kw OR (clinical trial):ti,ab,kw	720,816
12	10 OR 11	720,818
13	5 AND 9 AND 13	1803
